# The carbon footprint of transperineal prostate biopsy

**DOI:** 10.1002/bco2.70063

**Published:** 2025-07-30

**Authors:** Daniel A. Carson, Ali Hooshyari, Jesse Gale, Greg Evans, Flavio V. Ordones, Lodewikus P. Vermeulen

**Affiliations:** ^1^ Department of Urology Tauranga Hospital New Zealand; ^2^ Department of Surgery University of Auckland New Zealand; ^3^ Department of Surgery & Anaesthesia University of Otago Wellington New Zealand; ^4^ Toi Te Ora Public Health National Public Health Service New Zealand

**Keywords:** sustainability, carbon footprint, prostate biopsy, prostate cancer

## Abstract

**Objective:**

To evaluate the carbon footprint of transperineal prostate biopsy (TPPB). Climate change is the biggest global public health threat of the 21st century. Healthcare contributes 5% to global greenhouse gas emissions. Despite growing enthusiasm for sustainable urology, there is little data on the environmental impact of urological practice.

**Patients and methods:**

Emissions associated with TPPB (under local anaesthesia) at a hospital in Aotearoa New Zealand were estimated from electricity consumption, procurement of equipment/supplies, travel of staff and patients, waste disposal and sterilisation of linen. Emissions coefficients were used to determine CO_2_ equivalents (kgCO_2_e) emitted.

**Results:**

TPPB was associated with 70 kgCO_2_e of emissions per case. This equates to 280 km of travel by car, or an economy seat on a 70‐minute flight. The largest contributors were procurement (76%) and travel (23%). Electricity, waste disposal and sterilisation of linen did not contribute significantly to emissions (cumulatively <1.5%).

**Conclusions:**

This is the first study to evaluate the carbon footprint of a TPPB. Emissions were derived mostly from procurement and travel. These may be mitigated by review of standardised equipment packs, transitioning to reusables and introducing outreach biopsy clinics. Adherence to pragmatic evidence‐based guidelines for prostate cancer may reduce emissions associated with overdiagnosis and unnecessary biopsies. Further research is required to characterise the broader environmental impact of urology services.

## 1 INTRODUCTION

Climate change is the biggest global public health threat of the 21st century.^1^ Significant morbidity and mortality directly related to climate change are anticipated, including an additional 250,000 deaths per year between 2030 and 2050 on the current trajectory.^2^ Climate change must be mitigated through reductions in greenhouse gas emissions. Healthcare produces 5% of global emissions,^3^ of which surgical services represent a major component.^4^ Enthusiasm for “greening” surgical services is building, with recent pleas for immediate climate action across surgical units.^5^ Guidance documents on sustainable surgical practice are abundant, including the landmark Green Surgery Report.^5^ However, there is a paucity of primary research to inform evidence‐based recommendations.

‘Life cycle analysis’ (LCA) is more commonly being used to quantify the carbon footprint of a surgical procedure or device, although published LCA represent only a small fraction of procedures performed and devices used.^6^ Within urology, the environmental impact of ureteroscopes and cystoscopes has been studied,^7^, ^8^ and LCA of transrectal ultrasound (TRUS) guided prostate biopsy and radical prostatectomy.^9^, ^10^ In this study, we quantify the emissions associated with transperineal prostate biopsy (TPPB).

Prostate biopsy is well established as a pillar of prostate cancer diagnosis, and is one of the most common urological procedures with >1 million performed per year in the United States.^11^ TPPB has emerged in recent years as the gold standard for prostate biopsy, supported by European Association of Urology (EAU) guidelines.^12^ This is owing to superior detection of apical and anterior lesions, and reduced rates of re‐biopsy, infections and sepsis compared to TRUS biopsy.^11^, ^13^, ^14^ This study aimed to measure the carbon footprint of TPPB at Tauranga Hospital in Aotearoa New Zealand (AoNZ).

## 2 PATIENTS AND METHODS

This study was approved by the National Ethics Committee of New Zealand (ref 2022‐206). Written informed consent was obtained from patients. At Tauranga Hospital 200–250 TPPB are performed each year with a standardised equipment pack,^15^ using free‐hand technique with a needle guide (PrecisionPoint; Perineologic, MD, USA) under local anaesthesia (LA) in a clinic room by one urologist assisted by two nurses. Most patients have multiparametric prostate MRI prior to biopsy, as such most TPPB include lesion‐targeted and systematic core sampling.

### 2.1 Included sources of emissions

All LCA studies require selection of which sources of emissions will be included. We measured: (1) electricity consumption of the procedure room, (2) production and supply of equipment, disposables and pharmaceuticals used in the procedure (procurement), (3) travel of staff and patients, (4) waste disposal and (5) sterilisation of linen. We did not measure emissions related to: pathology services (an external provider), building construction, repeatedly used capital items such as the ultrasound machine, other associated consumption of food, stationery or information technology services (negligible in previous LCA studies). We did not include emissions associated with clinic visits and investigations before TPPB, treatment after TPPB or management of complications from TPPB (which are rare).

### 2.2 Estimation of emissions

We recorded 38 consecutive TPPB cases from December 2023. Emission sources were measured as described below, and converted to emissions (measured in CO_2_ equivalents, kgCO_2_e) using emission coefficients (EC) as shown in Table 1. Where possible, EC from the New Zealand Ministry for the Environment were used, and all were derived from AoNZ sources (except for the alternative method for procurement emissions, as detailed below).

1. *Electricity consumption:* Previous studies have attributed a fraction of total facility electricity consumption to a procedure, based on floor area.^19^ At our centre, electricity monitoring was not granular enough to determine outpatient department consumption. Overall hospital consumption was available, although attributing a fraction to a clinic room based on floor area was considered too unreliable due to the range of clinical spaces (360 beds including intensive care, and operating rooms) and their relative energy intensities, and not being able to separate energy use by radiology. The specific energy use of the clinic room was calculated based on consumption of the ultrasound machine, lighting and heating, ventilation and air conditioning (HVAC) systems. Cautious estimates were made regarding duration of use for each case. After‐hours use of HVAC was included. Peripheral sources of electricity consumption were not included (e.g. waiting room, staff areas). Energy use (kWh) was converted to emissions using EC for the averaged AoNZ energy grid.^16^
2. *Procurement:* The equipment pack used at our centre was itemised,^15^ including pharmaceuticals (LA and antibiotic prophylaxis). Volume of LA used in each case was recorded. *Cost data* was used to estimate emissions. This was achieved using an EC for medical equipment and pharmaceuticals (based upon 2019 data, as such costs were converted to 2019 NZD).^17^ An alternative method was also used, based on the *weight of raw materials* within the equipment pack (e.g. plastic, paper, metal) and weight of pharmaceuticals. Of note, EC from AoNZ sources were not available for many raw materials. Freight of equipment via air and road was included. See Appendix 1 for further details, including specific EC used. The former method was felt to be most reliable (see discussion); these results are presented below where not otherwise specified.
3. *Travel of staff:* Mode of travel to work and home suburb were recorded for the 38 cases audited. All staff travelled by private car. Travel distances were calculated using Google Maps (Google, CA, USA), divided across a full day case‐load (eight) and emissions estimated using an EC based on performance of an average car in AoNZ.^16^ *Travel of patients:* Method of travel was recorded for the 38 cases audited: all patients travelled by private car. Addresses of 100 patients having consecutively undergone TPPB (up to December 2023) were retrieved from a prospectively‐maintained database and used to calculate average travel distance, and emissions calculated in the same fashion as staff assuming all patients travelled by private car. A larger sample was collected for patients compared to staff because travel distance was expected to vary considerably given the large geographic area serviced.
4. *Waste disposal:* Waste created during the 38 audited cases was segregated into sharps, medical waste and landfill; this was weighed using an electronic scale. Sharps and medical waste are autoclaved off‐site before being transported to landfill. Distinct EC were used for sharps/medical waste,^18^ and general waste (in landfills with gas recovery).^16^ Emissions from freight for each waste stream were estimated using distances between our centre, waste processing facilities and landfills.^16^
5. *Sterilisation of linen:* Linen used in each case was weighed. A custom EC was created, using data on resources consumed per kg of linen (electricity, natural gas, water supply and wastewater treatment) at industrial laundry facilities in North America.^20^ Consumption data were used with local EC for each of these resources to arrive at the custom EC.^16^ Emissions from freight of linen were estimated as above for waste.

**TABLE 1:** Greenhouse gas emissions coefficients.

Emissions source	Unit	EC (kgCO_2_e/unit)	Reference
**Electricity**	kWh	0.083	^16^
**Procurement**			
Medical supplies	$	0.175	^17^
Pharmaceuticals	$	0.183	^17^
**Travel**			
Private car	km	0.252	^16^
**Waste disposal**			
General waste to landfill ‡	kg	0.232	^16^
Sharps	kg	0.442	^18^
Medical waste	kg	0.442	^18^
Freight of waste by truck (long‐haul)	tkm	0.105	^16^
**Sterilisation of linen**			
Laundering	kg	0.338	*
Freight of linen by truck (urban)	tkm	0.390	^16^

EC: Emission coefficient.

^*^ *Custom EC created for this study, based on resources consumed at industrial laundry facilities in North America, and local ECs for each of these resources*.

## 3 RESULTS

**FIGURE 1:**
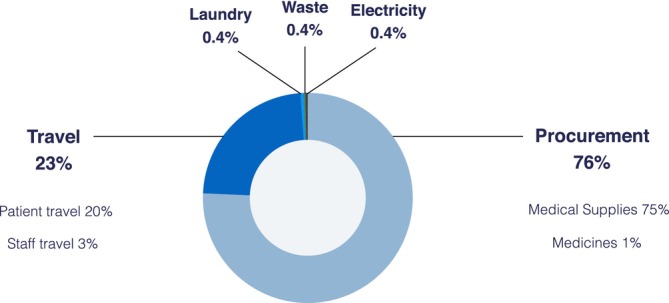
Contributions by emissions source to the carbon footprint (kgCO2e) of a transperineal prostate biopsy.

Emissions associated with a single TPPB were 70 kgCO_2_e. Contributions by emissions source are shown in Figure 1 and summarised in Table 2. Procurement comprised the largest component of emissions per case (76%), followed by travel (23%), mostly of patients (86% of travel emissions). Distance travelled by patients varied widely (return trip range 4–488 km), reflecting the large geographic area served by our department. Electricity consumption, waste disposal and sterilisation of linen cumulatively made up <1.5% of total emissions from TPPB. Waste produced was 731 g per case.

**TABLE 2:** Greenhouse gas emissions per transperineal prostate biopsy case.

Electricity consumption	*kWh*	*kgCO*_*2*_ *e*	*kgCO*_*2*_ *e (% total)*
Clinical space & equipment	3.3	0.27	
Total			**0.27 (0.4%)**
**Travel per case**	*Median km (range)*	*kgCO*_*2*_ *e*	*kgCO*_*2*_ *e*
Patient	56 (4–488)	14.11	
Staff	9 (8–16)	2.27	
Total			**16.38 (23.0%)**
**Procurement**	*$NZD (2023)*	*kgCO*_*2*_ *e*	*kgCO*_*2*_ *e*
Medical equipment	361.45	52.68	
Pharmaceuticals	5.44	0.82	
Total			**53.50 (76.0%)**
**Waste**	*Mean g ± SD*	*kgCO*_*2*_ *e*	*kgCO*_*2*_ *e*
Sharps	143 ± 3	0.06	
Medical waste	78 ± 41	0.03	
Landfill	511 ± 91	0.12	
Freight of waste		0.03	
Total	731 ± 101		**0.24 (0.4%)**
**Sterilisation of linen**	*Mean g ± SD*	*kgCO*_*2*_ *e*	*kgCO*_*2*_ *e*
Laundry	856 ± 141	0.29	
Freight of laundry		0.01	
Total			**0.29 (0.4%)**
**Total per case**			**70.41 kgCO**_**2**_ **e**

When using the alternative method used for procurement emissions (based on *weight of raw materials*), total emissions were estimated at 22 kgCO_2_e. Procurement emissions consisted of mostly freight (4 kgCO_2_e) rather than manufacture of the raw materials used (1 kgCO_2_e).

## 4 DISCUSSION

This study measured the carbon footprint of TPPB in AoNZ using LCA methods. TPPB was associated with 70 kgCO_2_e of emissions per case. This equates to 280 km of travel by car, or an economy seat on a 70‐minute flight.^21^

Procurement of supplies and equipment was the largest contributor to emissions (76%), in line with footprinting studies of other procedures.^19^, ^22^, ^23^ Review of the standardised equipment pack to minimise supplies used can be effective to reduce emissions, and transitioning to reusable alternatives where available. Of note, the European Society of Cataract and Refractive Surgeons have created a “sustainability index” calculator for ophthalmologists to assess their cataract surgery pack and be offered suggestions to improve the sustainability of their pack.^24^ A similar calculator for TPPB (or other commonly performed procedures) would be a useful resource for surgeons to benchmark their packs against a “green” standard. Given the number of prostate biopsies performed annually, even small changes to pack sustainability (including transitioning to reusables) are likely to have a material impact on emissions. This is likely to be more effective than common greening initiatives in hospitals, such as recycling and waste reduction, which typically do not have a significant impact on emissions.^25^ Where reusable alternatives are not available, reprocessing (sterilisation and reuse) of single‐use devices may play a role in reducing emissions.^26^

Travel contributed 23% to the carbon footprint of TPPB, most of which related to patient travel. Patients often travelled significant distances to their biopsy, with 20% of patients travelling >200 km round trip. Establishment of outreach biopsy clinics may reduce emissions, as well as improving access to biopsy. If all patients who underwent TPPB over the last two years at our centre (n = 415) were biopsied at their closest regional hospital, average travel distance would have been 44 km less. This equates to emission reductions of 11 kgCO_2_e (16%) per case. These findings also suggest that travel to pre/post‐biopsy appointments likely contributes significant emissions to the prostate cancer pathway at our centre. These could be mitigated by transitioning to virtual appointments where appropriate. Virtual urology clinics are acceptable among patients, deliver fiscal benefits in addition to environmental,^27^ and are associated with less patient costs, which may minimise non‐attendance.^28^ Where physical appointments are necessary, centres have successfully implemented “one‐stop” prostate cancer pathways including same‐day imaging and biopsy,^29^, ^30^ which can expedite diagnosis/treatment as well as reducing emissions from travel.

Waste did not significantly contribute to overall emissions (0.4%), consistent with other studies.^10^, ^19^, ^22^, ^23^ Although waste reduction and segregation for recycling is unlikely to give material reduction in emissions, other environmental impacts not measured in this study should not be overlooked. These include reduction of leachate from landfill waste, which leads to eutrophication and ecotoxicity,^31^ and minimising land used for landfill, which could be utilised for more sustainable purposes.

In addition to optimising sustainability of TPPB, efforts should be focused on minimising unnecessary or low‐value biopsies. Data suggests 40% of healthcare is wasteful, low‐value or harmful.^32^ This is at least loosely applicable to prostate cancer, where overdiagnosis and overtreatment are significant issues.^33^ EAU guidelines are increasingly focused to address this,^12^, ^34^ including strong recommendations to take into account life expectancy when considering eligibility for screening or offering a watchful waiting approach, and utilisation of MRI to avoid unnecessary biopsies. Modelling estimates that significant emissions could be mitigated by focusing on minimising low‐value care through a pragmatic evidence‐based approach to prostate cancer.^10^

No comparison of environmental impact has been made between TPPB/TRUS biopsy, although we propose TPPB is likely less carbon intensive. TPPB/TRUS do not drastically differ in terms of staff required, clinical setting, duration or equipment. However, TRUS biopsy is associated with higher rates of complications compared to TPPB, including sepsis (0.8% vs 0.1%).^35^ The environmental impact of these complications has not been assessed, although an admission for post‐TRUS biopsy sepsis costs $3000–19 000 (2019 USD),^36^ and one can safely assume this is associated with significant emissions.

To our knowledge, this is the first study to assess the carbon footprint of TPPB, with one other study evaluating TRUS biopsy. Leapman et al. estimated emissions associated with TRUS biopsy in the United States including pre‐biopsy MRI and pathology analysis.^10^ TRUS biopsy (excluding MRI and pathology) was associated with 33 kgCO_2_e (vs 70 kgCO_2_e in this study). Although insights can be gained by comparing these studies; differences in locality, included emissions sources and emissions estimation methods mean *comparing results at face value is problematic*. Leapman et al. found >50% of emissions were related to electricity consumption, versus <1% in our study (19 vs < 1 kgCO_2_e). This is due to >85% of electricity in AoNZ being generated by renewable sources. As such, advocating for sustainable electricity sources for hospitals will have a greater impact in nations with less renewable energy. In the TRUS LCA, there were less transport‐related emissions (11 vs 16 kgCO_2_e, for median round trip 25 vs 56 km). This may be explained by greater population density in the USA, and means implementing an outreach biopsy service may be less effective to mitigate emissions. The TRUS LCA reported substantially less emissions from procurement (4 vs 53 kgCO_2_e), which may be explained by supply chain differences between the USA and AoNZ (and differences in emissions estimation methods, see below). These comparisons highlight that overall carbon footprints from LCA cannot be compared at face value. Also, sustainability interventions will not be equally effective across regions; centres must consider local factors when deciding where to focus mitigation efforts.

A limitation of this study is the estimation of procurement emissions. We used two methods: based on *cost* and on the *weight of raw materials*, although both have their pitfalls. Cost does not reliably align with emissions^19^: sustainable items are not typically cheaper, and product price may reflect quantity discounts or other supplier arrangements. In the absence of more accurate local data to guide emission calculations (as encountered by other AoNZ authors^19^), we considered this first method the most reliable. Other international authors have used *weight* of medical equipment,^22^ or *weight of raw materials* (including Leapman et al). As an alternative method, we employed the latter. This ideally requires nation‐specific EC for each raw material, which are not widely available – most of the EC used in our alternative method were from international data. The procurement emissions estimate from this *weight‐based* method, even when taking into account long‐haul freight to AoNZ, was substantially less than the estimate based on *cost* (54 vs 5 kgCO2e). This *weight‐based* method likely understates the carbon footprint. Although it includes the emissions related to manufacture of each of the raw material components, it does not encompass the processing or assembly of these components into the products used. Ideally, medical supplies manufacturers would transparently and consistently report carbon footprints of products using LCA. Ongoing dialogue between surgeons, purchasers and suppliers is important to advocate for this. Until footprints are available for a range of products, using the cost of items presents a simple method to estimate emissions from procurement.

LCA in surgery is still in its infancy. There is a lack of consistent methodology, e.g. emissions sources included vary, with travel and procurement not routinely taken into account. Heterogeneity in methods and effects of study locality (explored above) mean that interpretation of LCA results is difficult, direct comparison of studies is problematic and erroneous conclusions about sustainability in surgery can easily be reached. A notable example is the environmental impact of single‐use versus reusable cystoscopes. Many LCA favour single‐use,^8^, ^37^, ^38^ although these studies have been criticised for their energy‐intensive sterilisation processes.^39^ Whereas another study that describes a more efficient sterilisation method suggests that reusable cystoscopes are far more environmentally friendly than single‐use (0.5 vs 2.4 kgCO_2_e).^40^ As such, readers in the space of surgical sustainability must be careful in appraising studies and their results/conclusions.

## 5 CONCLUSION

This is the first study to evaluate the carbon footprint of a TPPB. Emissions were derived mostly from procurement and travel. These may be mitigated by review of standardised equipment packs, transitioning to reusables and introducing outreach biopsy clinics. Adherence to pragmatic evidence‐based guidelines for prostate cancer may reduce emissions associated with unnecessary biopsies and overdiagnosis/treatment. This includes utilisation of MRI, which has revolutionised the workup of suspected prostate cancer. Modelling how MRI affects resource consumption and sustainability should be a priority for future research, along with characterising the broader environmental impact of urological services.

## AUTHOR CONTRIBUTIONS

DAC, JG, GE and LPV conceived the study. DAC and AH collected data. DAC analysed the data and wrote the manuscript. All authors edited and revised the manuscript and approved the final version for publication.

## CONFLICT OF INTEREST STATEMENT

DAC, AH, JG, GE, FVO and LPV have no relevant conflicts to declare.

## Supporting information


**Appendix S1.** Supporting Information.
