# Supplementary material for: The carbon footprint of transperineal prostate biopsy
Source: BJUI Compass. 2025 Jul 30;6(8):e70063. doi: 10.1002/bco2.70063 (PMC12310270; doi:10.1002/bco2.70063)
Supplement: Supplementary file 1 — Appendix S1. Supporting Information. [file BCO2-6-e70063-s001.docx]

**Appendix 1: Alternative method for procurement emission estimation, based on weight of raw materials in equipment pack.**

**Table A: Greenhouse gas emissions coefficients**

| *Emissions source* | *Unit* | *EC (kgCO_2_e/unit)* | *Reference* |
| --- | --- | --- | --- |
| **Equipment raw material** |  |  |  |
| Paper | kg | 1.28 | ^1^ |
| Plastics (general) | kg | 3.16 | ^1^ |
| Stainless steel (medical) | kg | 6.15 | ^2^ |
| **Pharmaceuticals** | kg | 4.5 | ^3^ |
| **Freight** |  |  |  |
| Air freight (long-haul) | tkm | 1.019 | ^4^ |
| Truck freight (long-haul) | tkm | 0.105 | ^4^ |

*EC: Emission coefficient.*

**Table B: Greenhouse gas emissions for procurement, by method.**

|  |  | *Emissions (kgCO_2_e)* | |
| --- | --- | --- | --- |
| **Procurement** |  | *Weight of raw materials method* | *Cost method* |
| Medical equipment |  | 1.05 | 52.68 |
| Freight |  | 4.03 |  |
| Pharmaceuticals |  | 0.13 | 0.82 |
| Total (% of total emissions per case) |  | **5.21 (23.5%)** | **53.50 (76.0%)** |
|  |  |  |  |

**References**

1. DEFRA. (2024). UK Government GHG Conversion Factors for Company Reporting. Department for Environment, Food & Rural Affairs. <https://www.gov.uk/government/collections/government-conversion-factors-for-company-reporting>
2. World Steel Association. (2021). Life Cycle Inventory Data and Methodology Report. Brussels: World Steel Association. <https://worldsteel.org>
3. Market Economics Ltd. (2023). Consumption Emissions Modelling: Report Prepared for Auckland Council. Auckland: Market Economics.
4. Ministry for the Environment. (2023). Measuring Emissions: A Guide for Organisations – 2023 Detailed Guide. New Zealand Government. <https://environment.govt.nz/publications/measuring-emissions-detailed-guide-2023/>
